# Robot-assisted therapy in stratified intervention: a randomized controlled trial on poststroke motor recovery

**DOI:** 10.3389/fneur.2024.1453508

**Published:** 2024-09-26

**Authors:** Yang Liu, Lijun Cui, Jixian Wang, Zihao Xiao, Zhi Chen, Jin Yan, Chuanxin M. Niu, Qing Xie

**Affiliations:** ^1^Department of Rehabilitation Medicine, Ruijin Hospital, School of Medicine, Shanghai Jiao Tong University, Shanghai, China; ^2^Department of Rehabilitation Medicine, Ruijin Rehabilitation Hospital, Shanghai, China; ^3^School of Medicine, Shanghai Jiao Tong University, Shanghai, China

**Keywords:** stroke, robotics, upper limb, motor recovery, stratified intervention

## Abstract

**Objective:**

To compare the effects of robot-assisted therapy with conventional therapy for accelerating stratified intervention in poststroke patients with upper limb dysfunction.

**Background:**

For stroke survivors, recovery of upper extremity function remains a major challenge in rehabilitation. Literature has suggested that the rate of recovery may improve if treatments can be individualized to their clinical profiles. However, there still lack clinical evidence on how to create treatment tailored to individual patients. Robot-assisted Therapy (RT) provides a straightforward approach to adjustment of the assistance-resistance continuum for individual patients. In early Brunnstrom stages of recovery, patients benefit from assistance training, whereas in later stages the training is favored with resistance. Therefore, RT may enhance Conventional Therapy (CT) but the use of RT in stratified intervention has not been investigated. This study evaluated the possible benefit of adopting RT following a protocol of upper-limb training, which was stratified with the Brunnstrom stage of each individual.

**Methods:**

This study was a single-blinded randomized controlled trial. A total of 53 patients with stroke were recruited and randomized into 2 groups (CT, *n* = 27, 3 dropped out and RT, *n* = 26, 2 dropped out). Both groups were trained once per day, 5 days per week for 4 weeks. The CT group received 30 min of conventional therapy; the RT group received 30 min of upper limb robot-assisted training. Patients were assessed at the beginning, week-2, and week-4 of the treatment. The outcome measures included the Fugl-Meyer Assessment Upper-Extremity (FMA-UE) and the Modified Barthel Index (MBI).

**Results:**

Across the 4-week intervention, participants in the RT group recovered 1.979 points of FMA-UE per week, compared to 1.198 points per week in the CT group (t_94_ = 3.333, *p* < 0.01); the recovery rate was 0.781 points/week higher in the RT group than in the CT group. Moreover, the recovery of FMA-UE was faster in proximal joints (t_94_ = 3.199, *p* < 0.01), and for patients in Brunnstrom Stage III (t_34_ = 2.526, *p* < 0.05). The improvements in MBI were not significantly different between RT and CT.

**Conclusion:**

Robot-assisted therapy showed initial evidence for the acceleration of post-stroke recovery of motor function in the upper limb. Initial observations suggested that patients in Brunnstrom recovery stage III might benefit the most from the stratified intervention assisted by robotics.

**Clinical trial registration:**

https://www.chictr.org.cn/showproj.html?proj=61834, Identifier [ChiCTR2000039010]. Registered 13 March 2020.

## 1 Introduction

Upper limb motor impairment is present in about 85% of stroke survivors (1). However, 30–60% of the cases may still show deficits in motor function after 6 months from the onset (2). Mounting evidence supports that motor recovery in the upper extremities is attainable using rehabilitation regimens, such as constraint-induced movement therapy, non-invasive brain stimulation, mental imagery, and bilateral arm training (3). However, one major challenge for upper-limb rehabilitation is that patients can be highly heterogeneous in the causes, locations, timing of stroke, etc. (4–6). As a result, clinical efficacy may be sub-optimal if the treatments fail to be tailored to the clinical profile of each patient (7).

One approach toward individualized treatment is stratified intervention, which subcategorizes patients into groups to apply group-specific treatments (8). A common indicator for stratification in stroke rehabilitation is the score of the Fugl-Meyer Assessment (9), which is a stroke-specific, performance-based impairment index. It is designed to assess motor functioning, balance, sensation, and joint functioning in patients with post-stroke hemiplegia (10). For example, in a study that gave 12-week training to patients with chronic stroke (11), those with moderate upper limb impairment (FMA-UE ≥ 26) gained 5.66 more points in wrist and hand compared with the other group (FMA-UE < 26). The advantage of using FMA as an indicator for stratification is that it contains standardized information about motor performance (10, 12). However, it is a challenge to associate a total score of FMA with a specific goal of post-stroke motor recovery (13). Alternatively, Brunnstrom Recovery Stages (BRS) provide a concise description of the key motor problems poststroke (14). Sum scores of the BRS could quickly provide an overall impression of a patient’s motor function as an alternative to inspecting the score of every joint. Moreover, sum scores could be an outcome indicator because any progress made on each item by a patient could be detected, which is useful for monitoring a patient’s overall change over time and determining the effects of intervention (15). Patients at Brunnstrom stages II, III, and IV (upper-limb) may all benefit from movement training (16–18), but in early stages, the movement needs substantial assistance (19), whereas in later stages the training is favored with resistance (20). For a heterogeneous collection of patients with stroke, therefore, their motor recovery may improve if the treatments can be stratified according to BRS (21).

Stratified intervention imposes new challenges on conventional therapies. On the one hand, differentiation in treatment plans must be rigorously followed across subgroups; on the other, within a subgroup, the treatment should be sufficiently consistent and repeatable. Robot-assisted therapy (RT) has the potential to facilitate stratified intervention, because of the high intensity, good repeatability, and task specificity provided by robotics (22–24). Given that the training may alter from assistive to resistive according to BRS, these requirements are straightforward to administer and regulate using programmable robots, which may enhance the eventual clinical outcome.

Previous studies have explored the key factors of robotic-assisted therapy for clinical efficacy, including the intensity (22), duration (23), and content of training (25). Data from several studies suggest that the recovery rate may improve if treatments can be individualized to their clinical profiles. A much-debated question is how to achieve functional progress according to individual heterogeneity, which may be the reason for the non-significant difference between groups in the RATULS (Robot-assisted training for the upper limb after stroke) study (26). Cases were limited, however, that incorporated robot-assisted therapy in stratified intervention. In a recent clinical study that stratified patients according to their FMA-UE, a combination of shoulder-elbow and wrist-hand robots was used to train chronic stroke patients, but the recovery was not significantly better in the robot-assisted group (11). In this study, we investigated whether upper-limb motor recovery could be accelerated by Robot-assisted Therapy, given that patients were subcategorized according to BRS. In the Robot-assisted Therapy (RT) group, participants performed upper-limb reaching movements using a robot with a group-specific setup of force; in the Conventional Therapy (CT) group, subjects accomplished comparable training under the guidance of an occupational therapist. We hypothesized that the recovery of motor functions in the upper extremities would be faster with RT. Results from this study may warrant larger-scale clinical trials for the evaluation of robot-assisted therapy for individualized treatments.

## 2 Methods

### 2.1 Participants

Patients were recruited between April 2020 and January 2021 from the in-patient rehabilitation center of Ruijin Hospital, School of Medicine, Shanghai Jiao Tong University, Shanghai, China. The data were also collected from the same in-patient rehabilitation center.

The inclusion criteria were:

1. Ischemic or hemorrhagic stroke confirmed by CT or MRI;
2. Age ranging from 18 to 80 years;
3. First onset of stroke within 1 ~ 12 months from recruitment;
4. Brunnstrom Recovery Stages (upper-limb) between 2 and 4;
5. Mini-Mental State Examination score > 15 and the ability to cooperate.

The exclusion criteria were:

1. Unstable medical condition;
2. Severe cognitive dysfunction;
3. Severe pain in the upper limb affecting the training;
4. Severe cardiac and pulmonary diseases;
5. Visual impairment;
6. Participation in other research involving robotic-assisted therapy.

All participants gave written informed consent before recruitment.

### 2.2 Study design and randomization

**Figure 1:**
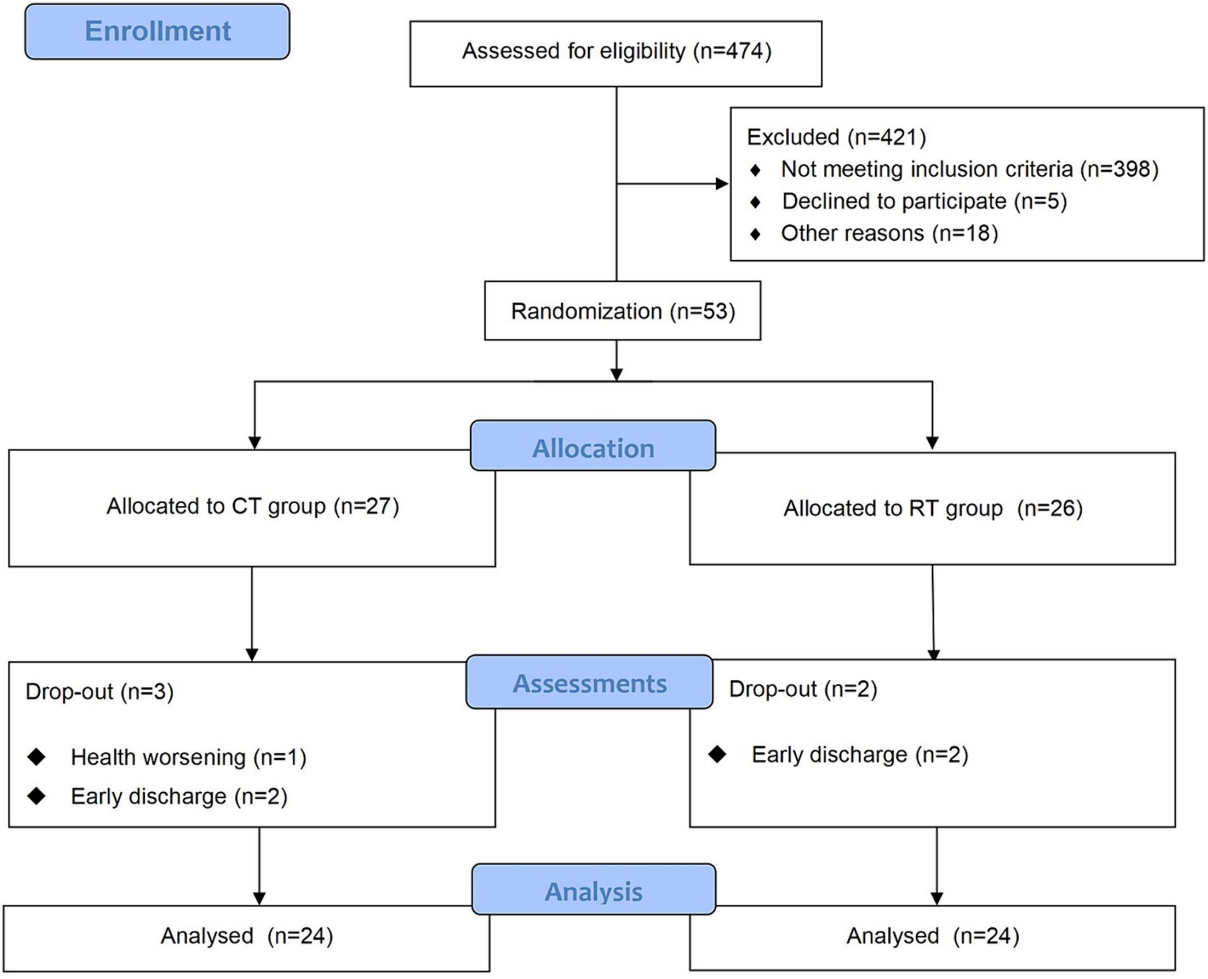
The CONSORT flow diagram of patient screening, randomization, assessment, and intervention.

**Figure 2:**
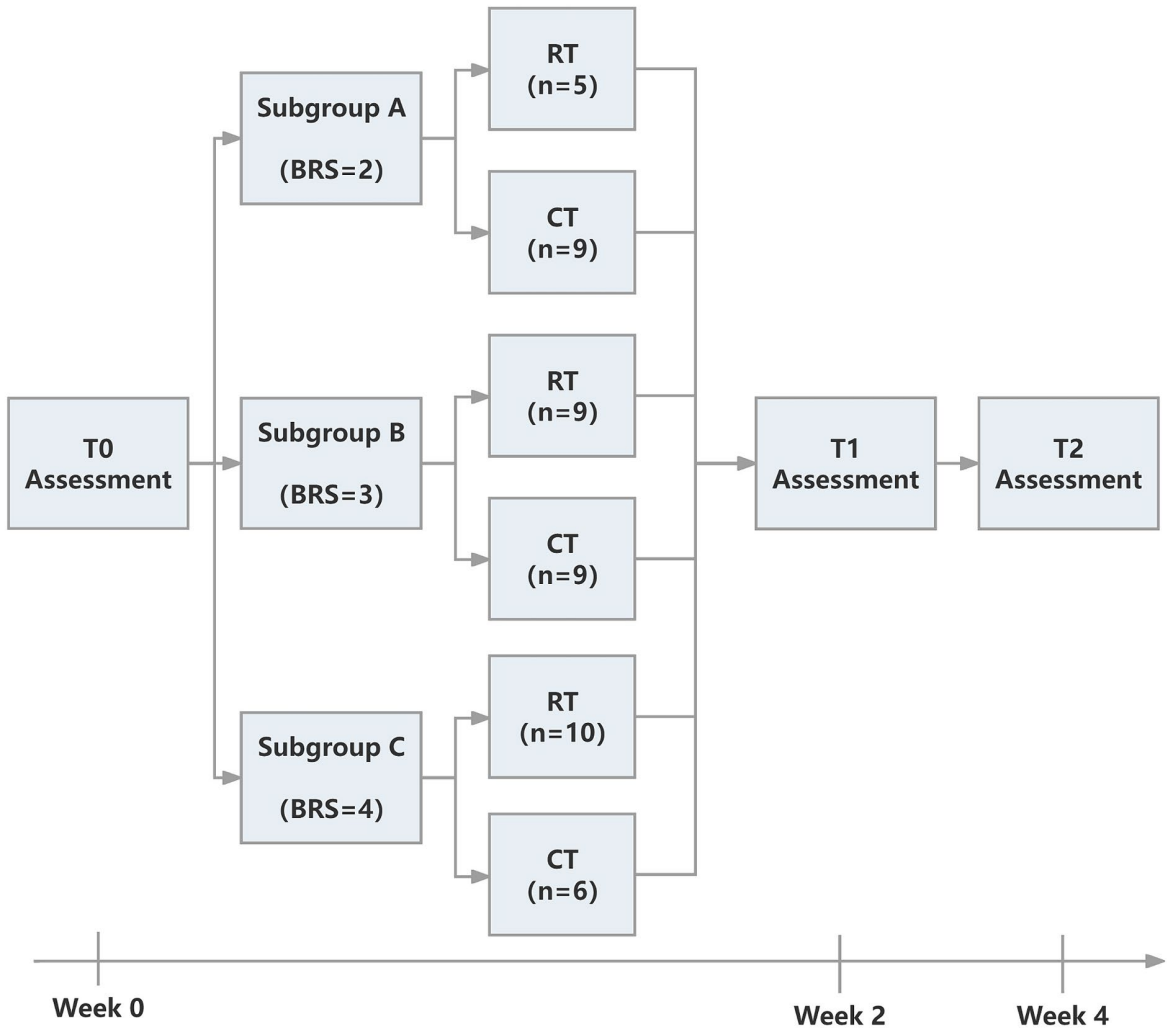
The intervention pipeline of two groups.

The study was approved by the Ethics Committee of Ruijin Hospital, School of Medicine, Shanghai Jiao Tong University. The trial was registered at the Chinese Clinical Trial Registry (ChiCTR2000039010, https://www.chictr.org.cn/showproj.html?proj=61834). The protocol was a single-blinded randomized controlled trial. We followed the CONSORT (Consolidated Standards of Reporting Trials) (27) statement and Figure 1 provides the flow diagram of patient screening, randomization, assessment, and intervention. All assessments were performed by an assessor who was blinded for group allocation. Participants were stratified by baseline Brunnstrom recovery stages (BRS II, III, or IV) and allocated to the RT group and CT group using block randomization with a size of 4, by a computer routine. Randomization was performed and documented by a researcher who was not involved in the intervention process. The allocation sequence was concealed from the researcher enrolling and assessing participants in sequentially numbered, opaque, sealed, and stapled envelopes. Therefore, the assessor was ignorant about the group to which the patient belonged. Each outcome measures were blinded to the assessor, therapists, and researchers until the final analysis. It took each participant around 4 weeks to accomplish the protocol (see Figure 2).

### 2.3 Interventions

The CT group received 30 min of conventional occupational therapy focused on upper-limb motor function; the RT group received 30 min of robot-assisted training with details to follow. In addition to the CT/RT interventions involved in this study, all participants also received routine therapies during the 4 weeks (5 days per week). The routine programs focused on motor impairment and recovery to establish deconditioning and fitness after stroke (28). There were several exercises guided by a physiotherapist in the routine therapy: balance training (sitting or standing), walking practice, aerobic exercises, strengthening exercises, and ADL training tailored to individual needs. Patients were required to rest for at least 5 min between exercises. The routine therapies took approximately 150 min each day for all patients. There was a 0.5 to 1-h break between intervention and routine physical therapy, and physicians would ensure patients had regained stamina before each intervention.

#### 2.3.1 Conventional therapy

**Figure 3:**
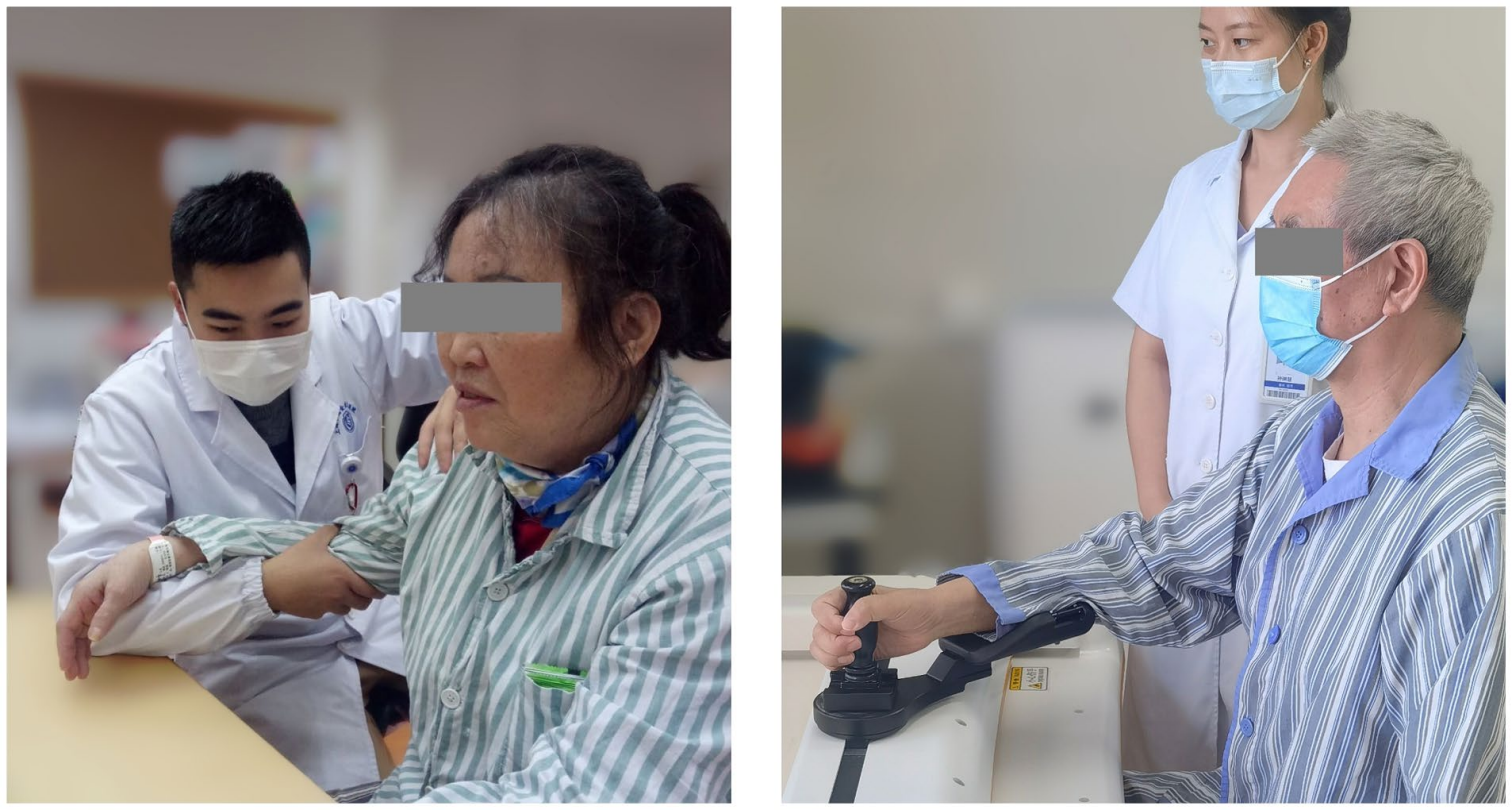
The actual scene of intervention during CT (left) and RT (right).

The CT involved 30-min arm activities on a table. During the training, the arm of the patient was held by a certified occupational therapist (Figure 3, left), and this therapist provided support, assistance, or resistance to all patients as required by the protocol:

Passive-dominated:

1. Supporting activities involving shoulder joints;
2. Simple activities involving shoulder and elbow joints;

Assistive-dominated:

1. Independent muscle training of upper extremity;
2. Basic grip training and simple movements;
3. Progressive grip training and simple activities with both hands;

Resistive-dominated:

1. Fine grip and upper-limb coordination activities;
2. Small palm muscle training and finger separation training;
3. Complex upper limb activities.

Correspondence between the types of training and subgroups is shown in Table 1.

**Table 1:** The intervention plan of two groups.

BRS of upper-limb	CT group	RT group
II	Mode: Passive-dominated, e.g., Passive anterior–posterior movements of the scapula.	Mode: Passive mode Velocity: 1–2 (0.025–0.05 m/s) Range: 22 cm*10 cm
III	Mode: Assistive-dominated, e.g., Elbow extension activities with the assistance of a therapist.	Mode: Assistive mode Velocity: 2–3 (0.05–0.075 m/s) Range: 31 cm*17 cm
IV	Mode: Resistive-dominated, e.g., Shoulder flexion movements with hands holding heavy objects.	Mode: Resistive mode Velocity: 3–5 (0.075–0.125 m/s) Range: 44ccm*28 cm

#### 2.3.2 Robot-assisted therapy

The RT session lasted 30 min supervised by a therapist (Figure 3, right). A commercial robot (ArmMotus M2, Fourier Intelligence Co. Ltd., Shanghai, China) was used to administer Robot-assisted Therapy. The robot provided a handle as the end-effector for participants to interact with. Participants would experience assistance or resistance through the handle. Before each session, the range of motion and specified amount of force were tested for each participant. During the gamified training session, icons of fruits and vegetables (radius 2 cm) appeared at random positions on the screen, one at a time. Patients were instructed in a sitting position to reach and stop at each icon for 1 s to acquire it. As can be seen, the gamified training session was equivalent to a series of point-to-point reaching movements. Simulated forces were added during the session with 3 different modes:

1. Passive mode: The handle guided patients to perform point-to-point reaching movements with a pre-defined velocity profile. The residual force applied by the patient was counteracted by the handle. Whenever the residual force on the handle exceeded a safety threshold (80 N), the robot would stop moving. The velocity was set to 0.025–0.05 m/s, and the range of motion in each movement was approximately 22 cm*10 cm. Thus the estimated number of repetitions during each RT intervention was around 150 times.
2. Assistive mode: An assistive force was determined for each patient before every session. The patient sat in front of the robot with the affected hand holding the handle, and then the patient was requested to make a 30 cm center-forward movement with a pre-defined assistive force. The initial magnitude of assistive force was set to 32 N, which usually meant accomplishing the task without any voluntary movement. If the patient was able to accomplish the task, then in the next trial the assistive force was reduced by 1 N. Eventually, the suitable magnitude of assistive force was set to the level that the patient was barely able to accomplish the task. Notice that the minimal assistive force was 15 N due to the design of the robot. During the search for a suitable magnitude of assistive force, at least a 1-min break was given between adjacent trials. The number of movement repetitions was around 100–180.
3. Resistive mode: A resistive force was determined for each patient prior to every session. The patient sat in front of the robot with the affected hand holding the handle, and then the patient was also asked to make a 30 cm center-forward movement with a pre-defined resistive force. The initial magnitude of resistive force was set to 1 N. If the patient was able to accomplish the task, then in the next trial the resistive force was increased by 1 N. Eventually, the appropriate magnitude of resistive force was set to the level that the patient was barely able to accomplish the task. Notice that the maximum resistive force was 5 N due to the design of the robot. During the search for a suitable magnitude of resistive force, at least a 1-min break was given between adjacent trials. The number of movement repetitions was also around 100–180.

The ArmMotus M2 robot also required specification of movement velocity prior to each session. Five levels of velocity were available: 0.025, 0.05, 0.075, 0.1, and 0.125 m/s, which represented the enforced average velocity during the passive mode, and recommended average velocity during the assistive and resistive modes. The assignment of force modes and velocity levels for subgroups is shown in Table 1. The scope of movement was measured for each participant prior to each session. The measurement program required the patients to reach as many points as possible, which were evenly scattered over the working space of the ArmMotus M2 robot. By fitting a rectangle encompassing all points that had been reached, the scope of movement could therefore be chosen as either small (22 cm*10 cm), medium (31 cm*17 cm), or large (44ccm*28 cm).

### 2.4 Outcome measurements

#### 2.4.1 Primary outcome measure

The primary outcome FMA-UE was evaluated every 2 weeks. FMA-UE was chosen due to its prevalence in clinical studies for stroke rehabilitation (29). The FMA-UE examines reflex activity and synergic voluntary movements. The evaluation includes 33 items and could be classified into four subscales: shoulder/elbow, wrist, hand, and coordination/speed. We were interested in both the total score (full points = 66) and the subscales for shoulder/elbow, wrist, and hand (30).

#### 2.4.2 Secondary outcome measure

The secondary outcome Modified Barthel Index was also performed every 2 weeks. The full score of MBI is 100, which assesses 10 aspects of the activity of daily living. Parts of MBI are related to upper limb motor function, including grooming, bathing, feeding, and dressing, with a total score of 30 points (31).

### 2.5 Statistical analysis

Statistical analyses were performed using R (version 4.1.2). A power analysis was performed using ‘simr’ package in R (32), considering *β* = 0.2, and *α* = 0.05. An estimated effect size was set to 0.9 according to the previous studies (33, 34). It was suggested that 21 patients in each group would be sufficient to detect the desired change. Baseline comparisons between groups were conducted by the t-test or Chi-square test. Effects of intervention on outcome measures were fitted using linear mixed-effect models (R library lme4 v1.1–21) as follows:


$$\left\{\mathrm{Outcome}\;\mathrm{measures}\right\}\sim \mathrm{time}+\mathrm{robot}+\mathrm{time}*\mathrm{robot}+\left(1|\mathrm{subject}\right)$$

where time was treated as a continuous variable, which represented the number of weeks passed since the beginning of intervention; robot denoted whether the intervention was CT or RT; the term $\left(1|\mathrm{subject}\right)$ accounted for subject-specific intercepts due to repeated measures. The time∗robot represented the interaction between time and robot. *p* < 0.05 was considered statistically significant. Outcome measures include FMA-UE, subscales of FMA-UE, and MBI.

## 3 Results

We screened 474 patients and 53 eligible candidates agreed to participate. Using per-protocol analysis (35), a total of 48 participants (24 in the CT group, 24 in the RT group) finished the 4-week intervention, and 5 participants dropped out during the intervention due to health worsening, early discharge, etc. Consultations with a multi-disciplinary team confirmed that none of the dropouts were related to the intervention in this study. According to Table 2, there was no significant difference between BRS, FMA-UE, MBI, and MAS (*p* > 0.05) in the baseline assessment of the two groups. For more detailed demographic characteristics of each participant, see the Appendix. No adverse events or unintended effects were reported.

**Table 2:** Demographic and clinical characteristics at baseline of stroke participants in robot-assisted therapy (RT) and conventional therapy (CT) groups.

Characteristics	CT (*n* = 24)	RT (*n* = 24)	*p*-value
**Characteristics**			
Sex (male/female)	15/9	20/4	0.10
Age (years)	66.1 ± 6.9	65.8 ± 9.0	0.89
Type of stroke (ischemic/hemorrhage)	19/5	19/5	0.41
Dominant side (left/right)	2/22	1/23	0.99
Affected side (left/right)	13/11	15/9	0.56
Stroke onset (months)	3.5 ± 2.7	3.9 ± 2.5	0.62
Modified Ashworth Scale (MAS)	0.4 ± 0.1	0.7 ± 0.1	0.14
**Evaluation**			
Brunnstrom recovery stages scale (BRS 2/3/4)	9/9/6	5/9/10	0.14
Fugl Meyer Assessment-Upper extremity (FMA-UE)	15.4 ± 8.7	15.9 ± 8.6	0.84
Modified Barthel index (MBI)	56.2 ± 16.3	59.1 ± 13.7	0.51

### 3.1 Primary outcome measures

The mixed-effect linear model showed that in the CT group, the FMA-UE score increased by 1.198/week (t_94_ = 7.228, *p* < 0.01). The interaction between time and group was also significant (t_94_ = 3.333, *p* < 0.01), meaning that in the RT group, the FMA-UE score increased by an additional 0.781 per week (total rate in RT = 1.979/week). Overall, the RT group recovered 65% faster than the CT group. Table 3 presents average scores at each evaluation time point. It is noteworthy that in the RT group, the mean increase in FMA-UE (7.9 ± 4.8, Mean ± SD) exceeded 5.25, the minimal-clinically-important-difference (MCID) as previously reported (36). In contrast, the mean increase in FMA-UE in the CT group (4.8 ± 3.2, Mean ± SD) did not exceed the MCID. A total of 15 out of 24 subjects in the RT group and 11 out of 24 in the CT group improved their FMA-UE over the MCID.

**Table 3:** Outcome measures at baseline, week-2, and week-4 of the two groups.

Outcome measure	CT group			RT group		
	Week 0	Week 2	Week 4	Week 0	Week 2	Week 4
**Motor function**						
FMA-UE total	15.42 ± 8.74	17.79 ± 9.28	20.21 ± 9.64	15.92 ± 8.60	20.50 ± 9.81	23.83 ± 11.02
FMA-UE Shoulder & Elbow	11.88 ± 5.14	13.92 ± 5.08	15.79 ± 5.27	12.00 ± 5.31	15.75 ± 6.15	18.33 ± 6.81
FMA-UE Wrist	0.75 ± 1.62	0.75 ± 1.75	0.79 ± 1.74	0.83 ± 1.86	0.96 ± 2.05	1.08 ± 2.50
FMA-UE Hand	2.46 ± 3.12	2.83 ± 3.33	3.33 ± 3.67	3.08 ± 3.41	3.75 ± 3.69	4.42 ± 3.92
**Daily activity**						
BI-total score	56.21 ± 16.26	61.21 ± 13.70	64.67 ± 14.59	59.13 ± 13.71	61.79 ± 12.30	66.33 ± 11.82
BI-Involving upper limbs	15.83 ± 5.20	18.13 ± 3.68	19.33 ± 4.85	17.21 ± 4.49	19.25 ± 3.69	20.58 ± 3.04

We also found that the RT group recovered faster than the CT group when the shoulder-elbow scores were extracted from the total FMA-UE. In shoulder-elbow subscales (full points = 36), the CT group increased by 0.979/week (t_94_ = 7.332, *p* < 0.01), whereas the RT group increased by a total of 1.583 per week (significant interaction, t_94_ = 3.199, *p* < 0.01), which was 62% faster than the CT group.

The hand subscale of FMA-UE increased by 0.219/week (t_94_ = 3.154, *p* < 0.01) in the CT group, whereas the RT group increased at a higher rate of 0.333/week, but the between-group difference was non-significant.

### 3.2 Secondary outcome measures

The activity of daily living measured in MBI increased by 2.115/week in the CT group (t_94_ = 5.436, *p* < 0.01), but the additional increase in the RT group was not significant. Similar results were found in the MBI upper-limb subscale (full points = 30), which increased by 0.875/week (t_94_ = 4.197, *p* < 0.01), and no significant difference was found between the CT and RT groups.

### 3.3 Subgroup analysis

**Figure 4:**
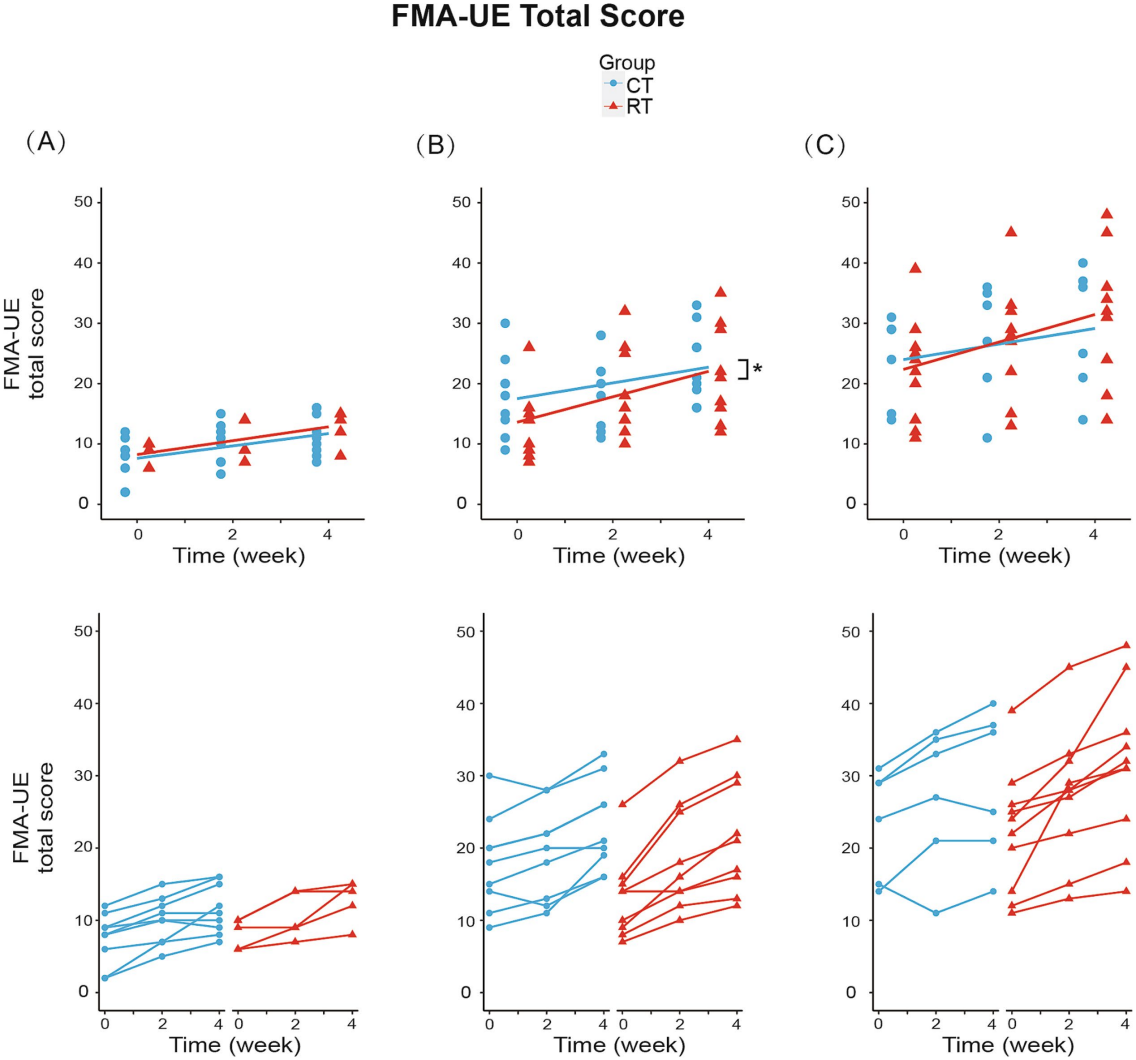
Change from baseline in FMA-UE total score in three subgroups. **(A)** In subgroup A (BRS = 2): RT group increased 1.150/week (*p* < 0.01), while CT group increased 1.028/week (*p* < 0.01). **(B)** In subgroup B (BRS = 3): RT group increased 2.111/week (*p* < 0.01), while CT group increased 1.306/week (*p* < 0.01). Between-group differences were significant (*p* < 0.05). **(C)** In subgroup C (BRS = 4): RT group increased 2.275/week (*p* < 0.01), while CT group increased 1.292/week (*p* < 0.01). The lines in the first row represent the arithmetic mean of the score. The lines in the second row represent the trend for the score of each patient in subgroups. *p < 0.05.

**Figure 5:**
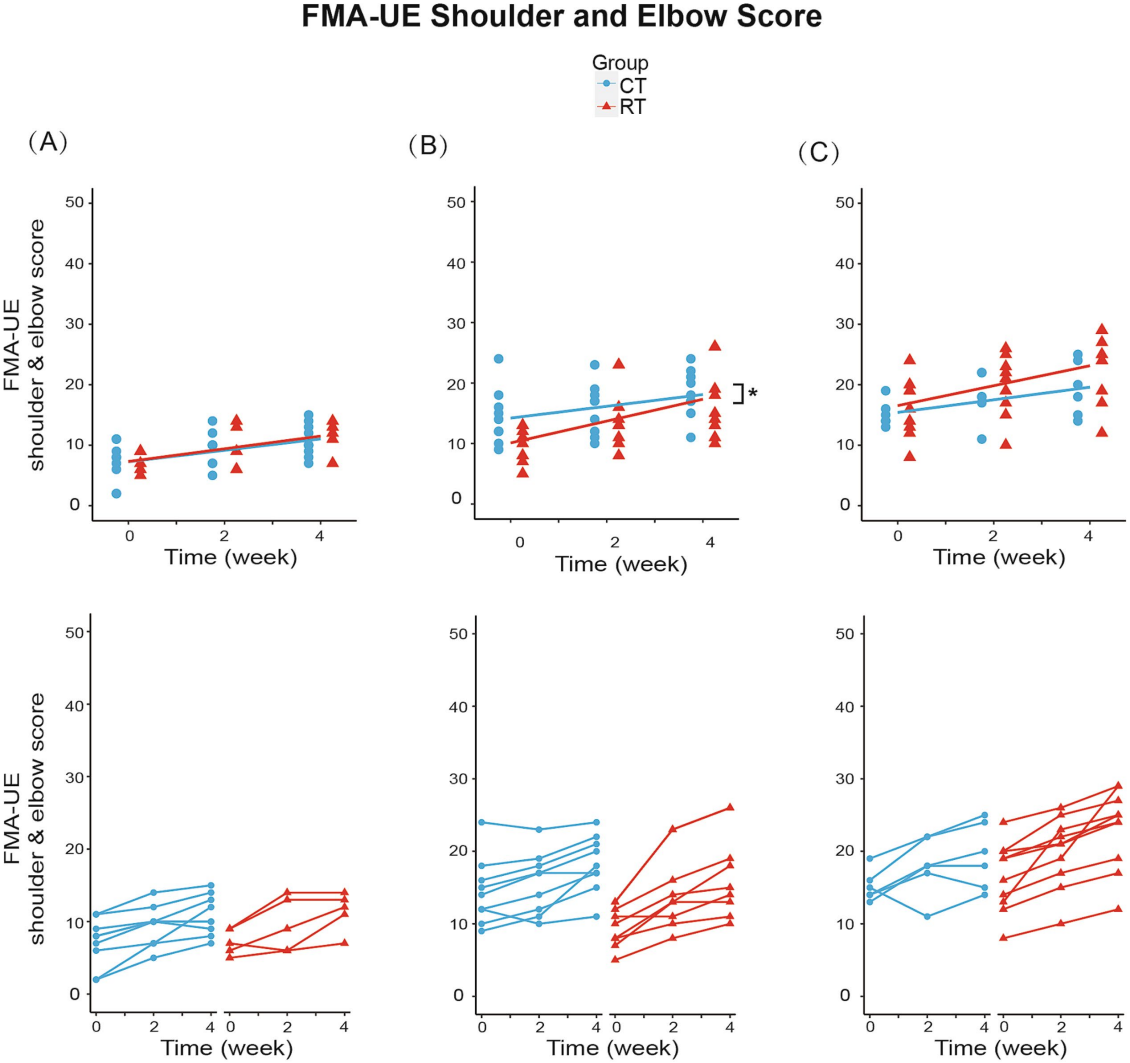
Change from baseline in FMA-UE shoulder and elbow score in three subgroups. **(A)** In subgroup A (BRS = 2): RT group increased 1.050/week (*p* < 0.01), while CT group increased 0.944/week (*p* < 0.01). **(B)** In subgroup B (BRS = 3): RT group increased 1.806/week (*p* < 0.01), while CT group increased 0.972/week (*p* < 0.01). Between-group differences were significant (*p* < 0.05). **(C)** In subgroup C (BRS = 4): RT group increased 1.650/week (*p* < 0.01), while CT group increased 1.042/week (*p* < 0.01). The lines in the first row represent the arithmetic mean of the score. The lines in the second row represent the trend for the score of each patient in subgroups. *p < 0.05.

We analyzed the effect of RT for each Brunnstrom subgroup. In subgroup A (BRS = 2), mixed-effect linear model found significant increase in FMA-UE total score (1.028/week, t_26_ = 6.144, *p* < 0.01, see Figure 4A), shoulder-elbow subscale (0.944/week, t_26_ = 5.543, *p* < 0.01, see Figure 5A), MBI (1.722/week, t_26_ = 2.918, *p* < 0.01) under CT, but no significant difference in between-group interaction was found in these outcomes, meaning that the RT did not incur faster motor recovery than did the CT for subgroup A.

In subgroup B (BRS = 3), the FMA-UE score increased by 1.306/week (t_34_ = 5.327, *p* < 0.01) under CT, compared to the 61% faster recovery of 2.111/week under RT (significant interaction, Figure 4B). Similarly, the shoulder/elbow FMA increased by 0.972/week (t_34_ = 4.168, *p* < 0.01) under CT, compared to an 86% faster recovery of 1.806/week under RT (t_34_ = 2.526, *p* < 0.05, see Figure 5B). The hand FMA and MBI scores showed no significant between-group interaction. Taken together, our results indicated that in subgroup B (BRS = 3), faster recovery of motor functions was observed under RT than CT.

In subgroup C (BRS = 4), FMA-UE total score (1.292 per week, t_30_ = 3.056, *p* < 0.01, see Figure 4C), shoulder-elbow subscale (1.042 per week, t_30_ = 3.627, *p* < 0.01, see Figure 5C), MBI (2.750 per week, t_30_ = 3.104, *p* < 0.01) were found significantly increased in the CT group, but the between-group interaction was not significant.

## 4 Discussion

Through this study, we found that both Robot-assisted Therapy and Conventional Therapy were likely to have a positive effect on post-stroke recovery of motor function. In comparison with conventional therapy, RT showed a higher rate of improvement in FMA-UE. Across all three subgroups, the added benefits of RT seemed the most prominent in patients with moderate motor impairment (BRS = 3). Taken together, our findings supported the hypothesis that even though both RT and CT groups received stratified intervention, the recovery of motor functions in the upper extremities would be faster with RT. Our results were consistent with these well-controlled RCTs, such as the RATULS trial, which demonstrated improvements in upper limb function within groups (26). However, our findings observed significant differences in the FMA scores between groups, which might be due to the stratified intervention or the different robot types. However, with a small sample size, caution must be applied, as our findings might not be extrapolated to all stroke patients.

A probable but noteworthy finding was that RT accelerated the motor recovery in the proximal joints of the upper limb (shoulder and elbow) but not distal ones (wrist and hand). Several possible explanations exist for this finding. Firstly, the participants moved the robot by contracting muscles around the shoulder and elbow, meanwhile, they kept the wrists and fingers strapped to the handle, therefore it could be the shoulder and elbow muscles that underwent the most training. It follows that the training ought to be extended to the wrist and hand, otherwise, it would leave a minimal chance for the wrist and hand to recover. Secondly, motor skills usually transfer from the proximal to the distal segments of the limb (37), thus the shoulder and elbow would lead the wrist and hand to show recovery. Thirdly, competition of adjacent joints in movement recovery suggests that proximal joints often recover better (38). In line with these findings, the end-effector robot employed in this study emphasized larger joint movements while fixing the hand at the terminal handle. In future studies, it might be possible to alter the type of robot to balance proximal and distal joints.

Another possible implication is that among the three subgroups, moderately impaired patients (BRS = 3, Subgroup B) recovered significantly faster when treated with RT compared to CT. In other two subgroups (BRS = 2 and 4) even though the between-group differences in FMA/week were not significant, the slopes were still steeper in RT, meaning that these two subgroups both contributed to the overall trend in pooled analysis. One reason why subgroup B (BRS = 3) outperformed the others was that patients in this stage might enjoy a higher capability of voluntary movement compared to subgroup A (BRS = 2). Therefore, the training may incur more afferent activity (i.e., the training signal) for motor re-learning (39). On the other hand, subgroups with better motor function (subgroup C, BRS = 4) might not be adequately challenged using the existing parameters, which inspires future studies on how much challenge is optimal for using robot-assisted therapy.

One limitation of this study was that it could not differentiate the contribution between assistive and resistive training, which required continuous monitoring of robot-applied force and the reaction from the participant. Note that the reaction might include a wide range of metrics, such as force, trajectory, electromyography, and brain activity. Another interesting but untested factor was whether the participants were compliant with the training, as has been shown critical for clinical outcome (40). Future robot-assistant studies could focus on finding more precise rehabilitation programs, such as the successful detection of voluntary muscle activity onset (41) and the utilization of brain-computer interfaces (BCI) system to achieve coordinated movements (42).

In clinic, our results support the application of robot-assisted therapy for the acceleration of post-stroke recovery in upper-extremity, especially with stratified intervention based on BRS. While this study was an initial, pilot study about the added benefits of robotics in stratified intervention for upper extremities poststroke. The clinical efficacy of our protocol was suggested by this pilot study, but it can only be asserted with larger-scale, multi-center trials.

## 5 Conclusion

Robot-assisted therapy showed promising clinical evidence in accelerating the poststroke recovery of upper-extremity motor performance following stratified intervention for 4 weeks. The improvements had been identified in general motor behaviors (Fugl-Meyer scores), especially in proximal parts of the upper limb. Among the tested (BRS II, III, and IV), individuals within Brunnstrom recovery stage III might benefit the most from robot-assisted training. However, with a small sample size, these findings cannot be extrapolated to all patients.

## Data Availability

The raw data supporting the conclusions of this article will be made available by the authors, without undue reservation.

## Supplementary material

SUPPLEMENTARY DATA SHEET 1 The detailed demographic characteristics of participants.
