# Supplementary material for: Robot-assisted therapy in stratified intervention: a randomized controlled trial on poststroke motor recovery
Source: Front Neurol. 2024 Sep 26;15:1453508. doi: 10.3389/fneur.2024.1453508 (PMC11464483; doi:10.3389/fneur.2024.1453508)
Supplement: SUPPLEMENTARY DATA SHEET 1 — The detailed demographic characteristics of participants. [file Data_Sheet_1.DOCX]

**Appendix**

Table S2 The detailed demographic characteristics of participants in the RT group.

| *Group* | *ID* | *Gender* | *Location of stroke* | *Affected* | *Brunnstrom* | *Age* | *Month* | *Plan* |
| --- | --- | --- | --- | --- | --- | --- | --- | --- |
| RT | S01 | M | Brainstem | Left | IV | 66 | 7 | C |
|  | S02 | M | Basal ganglia | Right | II | 61 | 3 | A |
|  | S03 | M | Periventricular area | Left | II | 68 | 2 | A |
|  | S04 | M | Basal ganglia | Left | III | 79 | 10 | B |
|  | S05 | M | Basal ganglia | Right | III | 78 | 7 | B |
|  | S06 | F | Parietal lobe | Left | III | 65 | 3 | B |
|  | S07 | M | Periventricular area | Right | IV | 65 | 4 | C |
|  | S08 | F | Basal ganglia | Right | III | 78 | 3 | B |
|  | S09 | M | Basal ganglia | Left | III | 64 | 3 | B |
|  | S10 | M | Thalamus | Left | II | 71 | 6 | A |
|  | S11 | M | Cortex | Left | IV | 68 | 2 | C |
|  | S12 | F | Cortex | Left | II | 68 | 7 | A |
|  | S13 | M | Cortex | Left | III | 71 | 1 | B |
|  | S14 | M | Basal ganglia | Left | IV | 53 | 1 | C |
|  | S15 | F | Periventricular area | Left | III | 60 | 1 | B |
|  | S16 | M | Basal ganglia | Left | II | 69 | 4 | A |
|  | S17 | M | Basal ganglia | Left | IV | 64 | 2 | C |
|  | S18 | M | Basal ganglia | Right | III | 43 | 3 | B |
|  | S19 | M | Centrum semiovale | Right | III | 66 | 2 | B |
|  | S20 | M | Basal ganglia | Right | IV | 44 | 4 | C |
|  | S21 | M | Brain stem | Right | IV | 66 | 4 | C |
|  | S22 | M | Periventricular area | Left | IV | 71 | 2 | C |
|  | S23 | M | Parietal lobe | Left | IV | 72 | 2 | C |
|  | S24 | M | Medulla | Right | IV | 69 | 4 | C |
|  | Mean |  |  |  |  | 65.8 | 3.9 |  |
|  | ±SD |  |  |  |  | ±9.0 | ±2.5 |  |

Table S3 The detailed demographic characteristics of participants in the CT group.

| *Group* | *ID* | *Gender* | *Location of stroke* | *Affected* | *Brunnstrom* | *Age* | *Month* | *Plan* |
| --- | --- | --- | --- | --- | --- | --- | --- | --- |
| CT | S01 | M | Basal ganglia | Left | IV | 61 | 9 | C |
|  | S02 | F | Cortex | Left | II | 56 | 2 | A |
|  | S03 | M | Periventricular area | Left | II | 72 | 2 | A |
|  | S04 | F | Basal ganglia | Left | II | 57 | 2 | A |
|  | S05 | M | Basal ganglia | Right | III | 70 | 2 | B |
|  | S06 | M | Basal ganglia | Right | III | 66 | 9 | B |
|  | S07 | M | Medulla | Left | III | 78 | 1 | B |
|  | S08 | F | Basal ganglia | Right | II | 66 | 5 | A |
|  | S09 | F | Medulla | Left | IV | 71 | 7 | C |
|  | S10 | F | Basal ganglia | Left | II | 54 | 2 | A |
|  | S11 | M | Cortex | Right | II | 66 | 3 | A |
|  | S12 | M | Periventricular area | Left | III | 74 | 1 | B |
|  | S13 | F | Medulla | Right | III | 65 | 3 | B |
|  | S14 | F | Basal ganglia | Right | III | 63 | 1 | B |
|  | S15 | M | Cortex | Right | II | 61 | 2 | A |
|  | S16 | M | Cortex | Left | IV | 67 | 3 | C |
|  | S17 | F | Cortex | Left | II | 61 | 5 | A |
|  | S18 | M | Periventricular area | Left | II | 76 | 4 | A |
|  | S19 | M | Medulla | Right | III | 67 | 10 | B |
|  | S20 | M | Basal ganglia | Right | II | 66 | 3 | A |
|  | S21 | M | Thalamus | Right | IV | 78 | 1 | C |
|  | S22 | M | Basal ganglia | Left | IV | 64 | 2 | C |
|  | S23 | F | Periventricular area | Right | IV | 72 | 4 | C |
|  | S24 | M | Cortex | Left | III | 56 | 4 | B |
|  | Mean |  |  |  |  | 66.1 | 3.5 |  |
|  | ±SD |  |  |  |  | ±6.9 | ±2.7 |  |
